# Transoral Robotic Surgery and Human Papillomavirus Infection: Impact on Oropharyngeal Cancer Prognosis

**DOI:** 10.3390/jcm13154455

**Published:** 2024-07-30

**Authors:** Jingtao Chen, Xing Zhang, Shida Yan, Xiyuan Li, Menghua Li, Ying Zhang, Shiting Zhang, Fengjiao Li, Ming Song

**Affiliations:** 1 Department of Head and Neck Surgery, Sun Yat-sen University Cancer Center, State Key Laboratory of Oncology in South China, Collaborative Innovation Center for Cancer Medicine, Guangdong Key Laboratory of Nasopharyngeal Carcinoma Diagnosis and Therapy, Guangzhou 510060, China; chenjt1@sysucc.org.cn (J.C.); zhangx1@sysucc.org.cn (X.Z.); yansd@sysucc.org.cn (S.Y.); liqy1@sysucc.org.cn (X.L.); limh1@sysucc.org.cn (M.L.); zhangying4@sysucc.org.cn (Y.Z.); zhangst1@sysucc.org.cn (S.Z.); 2 Department of Surgical Anesthesiology, Sun Yat-sen University Cancer Center, State Key Laboratory of Oncology in South China, Collaborative Innovation Center for Cancer Medicine, Guangdong Key Laboratory of Nasopharyngeal Carcinoma Diagnosis and Therapy, Guangzhou 510060, China

**Keywords:** oropharyngeal cancer, transoral robotic surgery, human papillomavirus, single-nucleotide variant, prognosis

## Abstract

**Background/Objective:** The incidence of oropharyngeal cancer (OPC) remains significant, with a rising prevalence of HPV-positive (HPV^+^) cases, underscoring the growing importance of appropriate treatment approaches for this condition. While HPV^+^ OPC typically exhibits a more favorable prognosis than HPV-negative (HPV^−^) OPC, certain HPV^+^ OPC patients still face adverse outcomes. This study aimed to assess the effectiveness of TORS versus traditional surgery in treating OPC patients and investigate the prognostic implications of specific variants in the HPV genome. **Methods:** The clinical information, including pathological features, treatments, and outcomes (death), of 135 OPC patients treated with traditional surgery from 2008 to 2018 (the non-TORS group) and 130 OPC patients treated with TORS from 2017 to 2021 (the TORS group) were obtained from Sun Yat-sen University Cancer Center (SYSUCC). A comparative analysis of 3-year overall survival (OS) was performed between these two groups. Furthermore, we conducted next-generation sequencing for the HPV16 genome of the 68 HPV^+^ OPC cases to characterize single-nucleotide variations (SNVs) in the HPV16 genome and evaluate its association with HPV^+^ OPC patient survival. **Results:** The comparative analysis of 3-year OS between the two groups (TORS vs. non-TORS) revealed a significant prognostic improvement in the TORS group for OPC patients with a T1–T2 classification (89.3% vs. 72.0%; *p* = 1.1 × 10^−2^), stages I–II (92.1% vs. 82.2%; *p* = 4.6 × 10^−2^), and stages III–IV (82.8% vs. 62.2%; *p* = 5.7 × 10^−2^) and for HPV^−^ patients (85.5% vs. 33.3%; *p* < 1.0 × 10^−6^). Furthermore, three SNVs (SNV1339A>G, SNV1950A>C, and SNV4298A>G) in the HPV16 genome were identified as being associated with worse survival. These SNVs could alter protein interactions and weaken the binding affinity for MHC-II, promoting viral amplification and immune evasion. **Conclusions:** TORS exhibited a superior prognosis to traditional surgery in OPC patients. Additionally, identifying specific SNVs within the HPV16 genome provided potential prognostic markers for HPV^+^ OPC. These significant findings hold clinical relevance for treatment decision-making and prognostic assessment in patients with OPC.

## 1. Introduction

Head and neck cancer is the sixth most common malignancy in the world, with more than 900,000 new cases and more than 450,000 deaths each year [1,2]. Oropharyngeal cancer (OPC) is a common type of head and neck cancer, with more than 100,000 new cases and more than 50,000 deaths worldwide every year [1]. Due to the increasing number of people infected with human papillomavirus (HPV), the incidence of HPV-positive (HPV^+^) OPC is rapidly increasing worldwide [3,4,5], most cases of which are caused by a single HPV type—HPV16 [6]—accounting for approximately 60–80% of all OPCs [7,8,9]. Similarly, HPV is a significant factor in the development of cervical cancer (CC), with HPV^+^ CC exhibiting particular clinical and epidemiological characteristics [10]. HPV^+^ CC patients may face persistent infection after surgical treatment, highlighting the complex interplay between HPV status and cancer management [11].

As an important prognostic factor for survival, HPV^+^ OPC patients have a better prognosis than HPV-negative (HPV^−^) OPC patients [12]. Despite high survival rates, traditional treatments, such as extensive resection and radiotherapy, for HPV^+^ OPC can cause many problems, including chronic pain, impaired speech functions, and difficulties in swallowing [13]. In recent years, surgical treatment has played a prominent role in the multidisciplinary diagnosis and treatment (MDT) of HPV^+^ OPC, especially the emergence of transoral robotic surgery (TORS), which affects the comprehensive treatment plan and prognosis of HPV^+^ OPC [14,15]. Studies have shown the favorable therapeutic benefit of TORS in OPC treatment, providing a minimally invasive approach that enhances surgical precision and reduces the recovery time, especially for HPV^+^ OPC. Nevertheless, it remains controversial [16]. Compared with traditional surgery, TORS improves postoperative outcomes and preserves important functional abilities, contributing to an overall better quality of life for patients. In addition, it is crucial to compare the postoperative survival outcomes of TORS with those of traditional surgery in OPC treatment.

Meanwhile, considerable clinical research has focused on treatment de-escalation, such as lowering the dose of radiation therapy or improving surgical techniques to reduce treatment-associated problems [17,18,19,20]. A significant barrier to the adoption of de-escalated treatment protocols for HPV^+^ OPC is that few predictors of poor prognosis exist. Furthermore, not all patients with HPV^+^ OPC benefit from current treatment strategies and achieve a good prognosis [19,20]. Experts are working to find clinically available markers for HPV^+^ OPC prognosis [21]. Integrating the HPV genome into the host cellular genome results in the expression of the E6 and E7 viral oncoproteins, causing the degradation of p53 and functional inactivation of Rb [22,23]. However, no studies have explored whether genetic variations within the HPV genome and functional mutations in the corresponding proteins can explain the heterogeneity of clinical behavior and prognosis between HPV^+^ OPC patients. Thus, it may be a valuable research direction to characterize the genetic variations in the HPV genome and evaluate their association with HPV^+^ OPC patient survival.

We selected 265 cases of OPC with more than 3 years of follow-up, including 135 OPC patients treated with traditional surgery (the non-TORS group) and 130 OPC patients treated with TORS (the TORS group) to explore the effect of TORS on the prognosis of patients with OPC and investigate the genetic heterogeneity of the HPV genome in HPV^+^ OPCs. Moreover, we conducted next-generation sequencing (NGS) to characterize the single-nucleotide variations (SNVs) in the HPV16 genome from 68 HPV16^+^ OPC tissues. Here, we present the results regarding the prognosis of OPC patients. We aimed to investigate the efficacy of TORS in OPC and the prognostic value of genetic variations in the HPV 16 genome in HPV^+^ OPC.

## 2. Materials and Methods

### 2.1. Study Population

Initially, we conducted a retrospective screening of the medical health records of OPC patients who underwent traditional surgery in the Sun Yat-sen University Cancer Center (SYSUCC) between 2008 and 2018 (the non-TORS group). A total of 613 medical health records were consulted, and 478 were excluded due to prior cancer histories (*n* = 101), prior cancer treatments (*n* = 248), incomplete follow-up records (*n* = 70), or anatomical location updates (*n* = 59), resulting in a non-TORS group of 135 cases (Figure S1). Additionally, a retrospective screening of the medical health records of OPC patients who underwent TORS in the SYSUCC from April 2017 to November 2018 was performed (the TORS group). Out of 250 records consulted, 180 were for OPC, with 50 excluded due to prior cancer histories (*n* = 8), prior cancer treatments (*n* = 10), or less than 3 years of follow-up (*n* = 32), resulting in a TORS group of 130 cases (Figure S2). Finally, we screened 265 cases from both the TORS and non-TORS groups, of which 85 were HPV^+^ cases. These were further reduced to 68 samples that completed HPV16 whole-genome sequencing (the HPV16-WGS group; Figure S3) due to DNA extraction failures (*n* = 6), non-HPV16 positivity (*n* = 7), and low coverage and/or poor resolution (*n* = 4).

### 2.2. Clinical Data Abstraction

The clinical information, including pathological features, treatment responses, and outcomes (death) of 135 OPC patients who received traditional surgery from 2008 to 2018 (the non-TORS group) and 130 OPC patients who received TORS from 2017 to 2021 (the TORS group) were collected. The 8th edition American Joint Commission on Cancer (AJCC) staging guidelines was used to stage OPC patients with HPV detection, and the 7th edition of the AJCC staging guidelines was used to stage OPC patients without HPV detection. The clinicopathological characteristics of the two groups are shown in Table 1. The Kaplan–Meier method and log-rank test were used for survival analysis.

### 2.3. Specimen Collection, Processing, and DNA Isolation

The specimens used in this study were supplied by the Biospecimen Repository and the Department of Head and Neck Surgery, SYSUCC. A total of 68 HPV^+^ OPC frozen tissues derived from surgically treated patients with more than 3 years of follow-up data were included. All tissues were freshly frozen in liquid nitrogen within 30 min of resection. Genomic DNA was extracted from each sample using the FastPure Cell/Tissue DNA Isolation Mini Kit (DC102-01, Vazyme, Nanjing, China), following the manufacturer’s instructions. No more than 10 samples were processed in a single batch to minimize the possibility of cross-contamination.

### 2.4. Nested PCR Amplification and Genome Sequencing

The complete 8 kb genomes of the HPV16 isolates from the genomic DNA extracted from the clinical OPC specimens were amplified using overlapping PCR, as previously studied [24,25,26]. In brief, three sets of primers for nested PCR were applied to amplify the entire genomes in three overlapping fragments (Supplementary Table S1). For the overlapping PCR, the Green Taq Mix (P131, Vazyme, Nanjing, China) was used for the amplification, and the 0.5× VAHTS DNA Clean Beads (N411, Vazyme, Nanjing, China) were used to clean up. The amplified HPV16 DNA was sequenced with a next-generation sequencing (NGS) assay using the BGI MGIseq2000 sequencing platform. The quality control followed the SOAPnuke manual [27]. If multiple HPV16 variants were detected, the predominant variant was determined based on at least 60% of sequence reads. Five specimens were excluded due to poor read depth, incomplete coverage across the genome, or an inability to assign a lineage [26]. After assembling 40 OPC-HPV16 genomes, we used HPV16 (NC_001526.4) with the reference for sequence alignment with MAFFT (v7.310) [28]. The SNVs were generated with the SNP sites (v.2.5.1) [29], and the nucleotide variants were annotated with consequence using VEP (v.109.3) [30].

### 2.5. Protein Structure Prediction and Visualization

HPV16 E2 (PDB code: 1DTO) and E1 were used in this study. For HPV16 E1, we employed AlphaFold [31] (v2.3.2; running with 5 cycles) via the ColabFold webserver (https://colab.research.google.com/github/sokrypton/ColabFold/AlphaFold2.ipynb, accessed on 1 June 2024) for the protein structure prediction and selected the top-ranked protein structure for subsequent analysis. Before docking calculations, the structures of E1 and E2 were assigned appropriate protonation states via the PDB2PQR server [32]. ClusPro [33], a protein–protein docking server, was used in HPV16 E1–E2 docking simulations with the default parameter ‘balanced coefficient’. The whole structure was visualized using PyMol (v2.5; https://pymol.org/, accessed on 10 June 2024).

### 2.6. Affinity Prediction of MHC Binding to HPV Protein

We searched the previously identified immune epitopes of the HPV16 E1, E2, and L2 proteins at the IEDB [34] and extracted those covering our identified prognosis-associated SNVs. We used the “Epitope Analysis Resource” web portals provided by the IEDB [34] with default parameters to predict the binding affinity of MHC-I and MHC-II to the epitope peptides, carrying either the wild-type or mutated form of our identified SNVs. The sign test was used to compare the overall epitope-to-MHC affinity between the wild-type and mutated forms for each identified SNV.

### 2.7. Statistical Analyses

The patient characteristics were evaluated overall. The Cox proportional hazard models were used to evaluate the association between each HPV16 SNV and overall survival (OS). To assess the risk of death from any cause, the only censoring event was at the time of the last known follow-up, and the years since cancer diagnosis were used as the time variable. The proportion of risk explained by the effect of cancer-related SNVs was estimated in the validation sample. To assess the associations, the hazard ratios (HRs) with 95% confidence intervals (95% CIs) and *p*-values of cancer-susceptible SNVs were obtained with Fisher’s exact test using GraphPad Prism software v. 8.0.2. [35].

### 2.8. Ethical Approval

The study protocol was reviewed and approved by the Institutional Review Board of Sun Yat-sen University Cancer Center (no. B2022-116-Y02; date: 28 April 2024). The need for informed consent was waived per the IRB’s decision.

## 3. Results

### 3.1. Participant Characteristics

In total, 265 patients, including 135 cases receiving traditional resections (the non-TORS group) and 130 OPC patients with TORS (the TORS group), were selected (Table 1). For the whole cohort, the mean age at diagnosis was 56 years (range: 33 to 85), and the majority were male (M–F ratio: five). Over 65% of patients reported histories of tobacco use (55.5%; 147/265) or alcohol use (70.9%; 188/265). Notably, the TORS group had a significant enrichment of HPV-positive cases (52.3%; 68/130), compared with only 17 HPV-positive patients in the non-TORS group (41.5%; 17/41). This discrepancy arose from a lack of emphasis on HPV identification among OPC patients in our hospital before 2016, leading to 94 cases with unknown status of HPV infection in the non-TORS group. Correspondingly, due to the modification in the 8th edition of the TNM classification for HPV-positive OPC, most patients in the TORS group presented with stages I–II of the disease (77.7%, 101/130), while 22.3% presented with stages III–IV.

Sixty-eight patients received HPV16 whole-genome sequencing, and five specimens were excluded due to poor read depth, incomplete coverage across the genome, or an inability to assign a lineage. For the HPV16-WGS group (Supplementary Table S1), the OPC patients were mainly male (79.4%; 36/68), with an average age of 56 years old (from 33 to 70), most of which were staged at T1–T2 (89.7%; 61/68).

### 3.2. TORS Can Improve Prognosis of OPC

**Figure 1:**
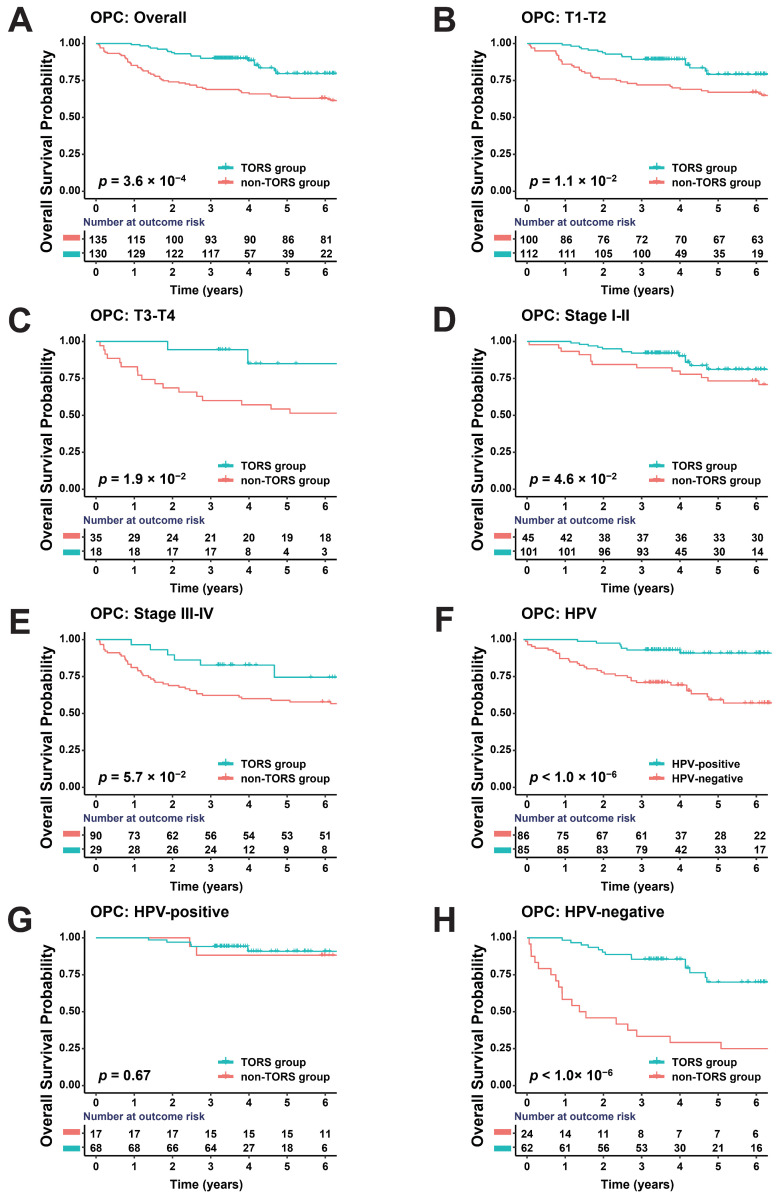
Kaplan–Meier plots for overall survival of different groups: (**A**) Overall survival probability comparing all OPC patients treated with traditional surgery (non-TORS group; red) and those treated with TORS (TORS group; blue). (**B**) Overall survival probability comparing OPC patients with T1–T2 treated with traditional surgery (non-TORS group; red) and those treated with TORS (TORS group; blue). (**C**) Overall survival probability comparing OPC patients with T3–T4 treated with traditional surgery (non-TORS group; red) and those treated with TORS (TORS group; blue). (**D**) Overall survival probability comparing OPC patients with stages I–II treated with traditional surgery (non-TORS group; red) and those treated with TORS (TORS group; blue). (**E**) Overall survival probability comparing OPC patients with stages III–IV treated with traditional surgery (non-TORS group; red) and those treated with TORS (TORS group; blue). (**F**) Overall survival probability comparing HPV-negative (red) and HPV-positive (TORS group; blue) OPC patients. (**G**) Overall survival probability comparing HPV-positive OPC patients treated with traditional surgery (non-TORS group; red) and those treated with TORS (TORS group; blue). (**H**) Overall survival probability comparing HPV-negative OPC patients treated with traditional surgery (non-TORS group; red) and those treated with TORS (TORS group; blue).

After a median follow-up period of 4.2 years and 6.7 years, 26 and 52 deaths were reported in the TORS and non-TORS groups, respectively. Overall, the 3-year OS of the TORS group was 90.0%, which was significantly better than that of the non-TORS group (68.9%; *p* < 0.05; Figure 1A). For OPC patients with local–early classification (T1–T2), the 3-year OS of the TORS group was better than that of the non-TORS group (89.3% vs. 72.0%; *p* = 1.1 × 10^−2^; Figure 1B). For patients with a local–advanced classification (T3–T4), a favorable 3-year OS was also revealed in the TORS group, although no significant difference was found compared with the non-TORS group (94.4% vs. 60.0%; *p* = 1.9 × 10^−2^; Figure 1C). Furthermore, the 3-year OS was better in the TORS group than in the non-TORS group for both stages I–II (92.1% vs. 82.2%; *p* = 4.6 × 10^−2^; Figure 1D) and III-IV OPC patients (82.8% vs. 62.2%; *p* = 5.7 × 10^−2^; Figure 1E).

Previous studies have found that the overall prognosis of HPV^+^ OPC is better than HPV^−^ OPC [36,37], which was verified in our study (92.9% vs. 70.9%; *p* < 1.0 × 10^−6^; Figure 1F). In addition, the TORS group demonstrated a better 3-year OS than the non-TORS group for both the HPV^+^ OPC (94.1% vs. 88.2%; *p* = 6.7 × 10^−1^; Figure 1G) and HPV^−^ OPC groups, especially HPV^−^ OPC patients (85.5% vs. 33.3%; *p* < 1.0 × 10^−6^; Figure 1H).

### 3.3. Some SNVs of HPV16 Genome May Affect OPC Prognosis

**Figure 2:**
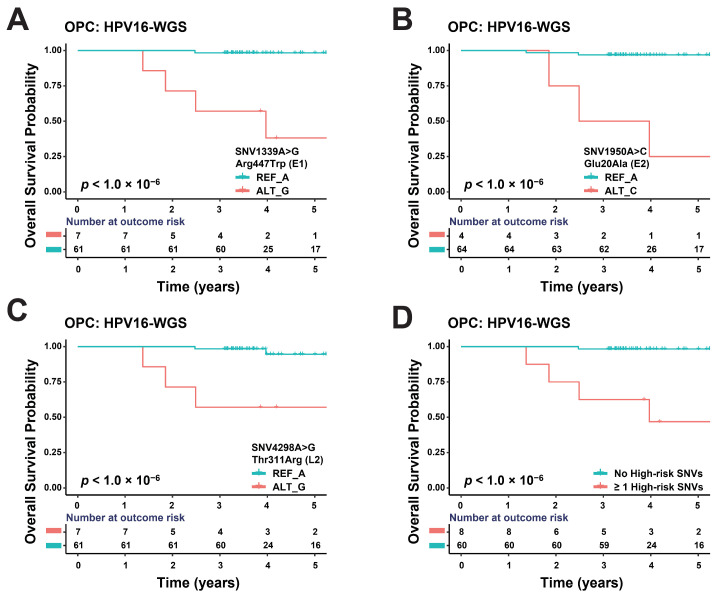
Kaplan–Meier plots for overall survival of individuals with high-risk HPV16 SNVs and comparing patients without and with at least one high-risk HPV16 SNV. Overall survival probability comparing the alternative allele (red) with the reference allele (blue) for the following SNV positions within the HPV16 genome: (**A**) SNV1339A>G; (**B**) SNV1950A>C; and (**C**) SNV4298A>G. (**D**) Overall survival probability comparing patients with at least one high-risk HPV16 SNV (red) and patients with no high-risk HPV16 SNVs (blue).

Among the forty tumor tissues included in our genomic analyses (the HPV16-WGS group), the median follow-up time was 4.13 years (1.37–7.10 years) with five patients who died (7.4%; median time from diagnosis to death: 2.43 years, 1.37–3.96 years). For the HPV16-WGS group, we identified three single-nucleotide variants (SNVs) among the forty HPV16 genomes with a minor allele frequency (MAF) of 1.0% or greater and evaluated the correlation between each of these three HPV16 SNVs and OS (Table 2). These three SNVs were localized to three regions within the HPV16 genome—the E1 gene (SNV1339A>G), E2 (SNV1950A>C), and L2 genes (SNV4298A>G)—which were strongly associated with an increased hazard of death (*p*_SNV1339A>G_ < 1.0 × 10^−6^, HR_SNV1339A>G_: 6.08 × 10^3^; *p*_SNV1950A>C_ < 1.0 × 10^−6^, HR_SNV1950A>C_: 3.10 × 10^4^; *p*_SNV4298A>G_ < 1.0 × 10^−6^, HR_SNV4298A>G_: 5.23 × 10^2^; Table 2; Figure 2A–C). Among the 40 HPV^+^ OPC patients, 8 (11.8%) had at least one of the three high-risk HPV16 SNVs. Patients with at least one of the three high-risk HPV16 SNVs identified had a median survival time of 3.61 years compared with 4.20 years for patients without any high-risk HPV16 SNVs (*p* < 1.0 × 10^−6^; HR = 1.29 × 10^3^ (95% CI: 7.67 × 10^1^ to 2.16 × 10^4^); Figure 2D), suggesting that these SNVs have a strongly negative prognostic value for OPC patients.

### 3.4. Prognosis-Associated SNVs May Alter Interactions and Functions between Proteins

**Figure 3:**
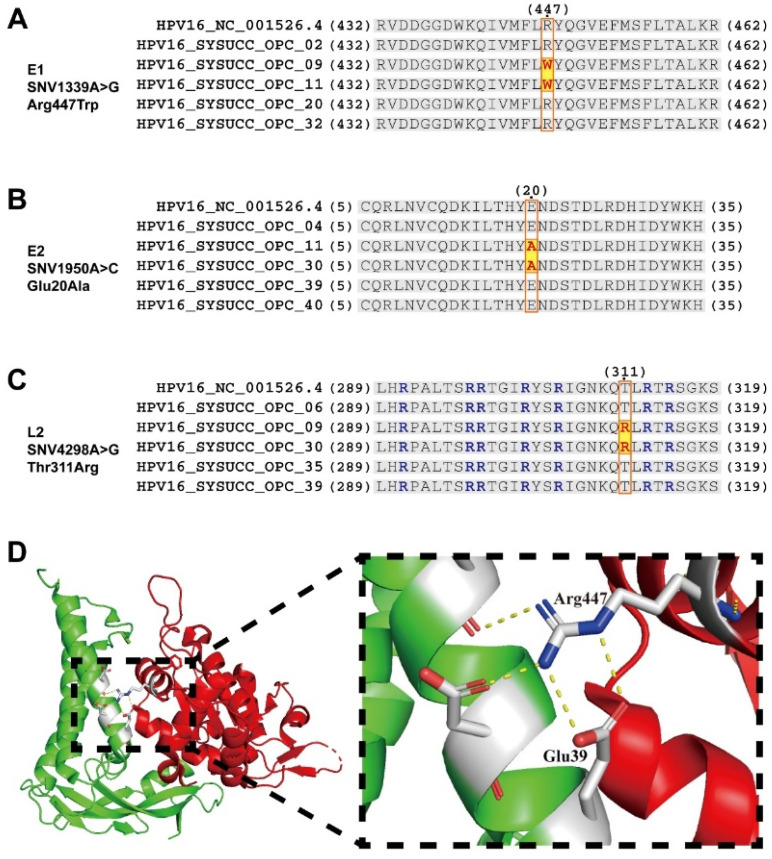
The alternation of interactions and functions between proteins corresponding to prognosis-associated SNVs: (**A**) Amino acid sequence alignment of E1 proteins 432–462; the mutation R447W (shaded in yellow and highlighted in red) within the E1 protein binding to the E2 protein Glu39 may enhance E1–E2 binding. (**B**) Amino acid sequence alignment of E2 proteins 5–35; the mutation E20A (shaded in yellow and highlighted in red) within the E2 protein may enhance E1–E2 binding. (**C**) Amino acid sequence alignment of L2 proteins 289–319, a region rich in arginine (highlighted in blue); the mutation T311R (shaded in yellow and highlighted in red) led to the addition of an arginine to this region, which may indicate the nuclear localization and nuclear retention of L2. (**D**) Structure of predicted HPV16 E1–E2 complex. The amino acid residues at the interface between E1 (red) and E2 (green) are colored grey (Arg447 in E1 and Glu39 in E2). Hydrogen bonds are represented as yellow dashed lines.

Given that SNVs within the HPV16 genome may affect the prognosis of HPV^+^ OPC, we investigated the possible changes in interactions and functions between the proteins caused by each SNV. According to previous studies, Arg447 within the E1 protein binds to Glu39 of the E2 protein [38]. Furthermore, the amino acid mutation from arginine to the tryptophan of A447W in E1 (SNV1339A>G; Figure 3A) may increase the binding ability to Glu39 within E2 [39], thereby enhancing E1–E2 binding, which promotes viral replication and leads to poor outcomes in OPC patients. The SNV1950A>C within the E2 gene led to the amino acid mutation E20A (Figure 3B) and enhanced E1–E2 binding [40], which, in turn, promoted viral replication and led to a poor prognosis.

Furthermore, SNV4298A>G resulted in the amino acid mutation T311R of the HPV16 L2 protein (Figure 3C), which may enhance the nuclear localization and nuclear retention of L2 and promote the integration of the HPV genome into the host genome [41], thus affecting the prognosis of patients with OPC.

### 3.5. Prognosis-Associated SNVs May Alter Protein Affinity for MHC-II Binding

**Figure 4:**
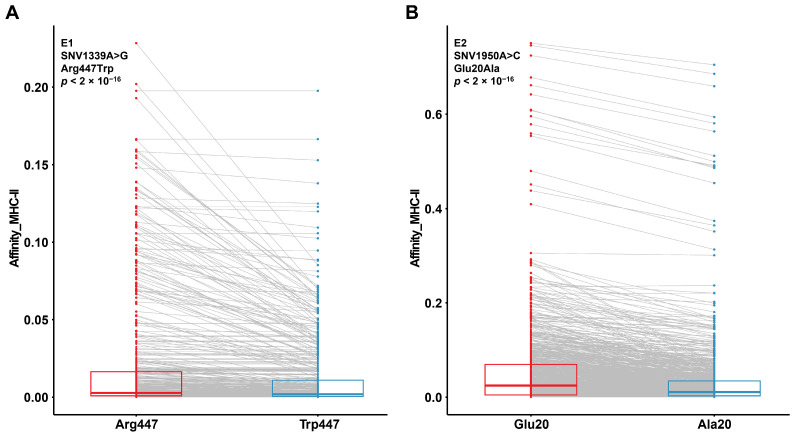
The MHC-II binding affinity comparison of epitopes carrying the wild-type and mutated forms of prognosis-associated HPV16 SNVs in E1 (**A**) and E2 (**B**).

Aside from the intrinsic effects on maintaining and strengthening viral activities, these prognosis-associated SNVs can also help HPV16 reach higher pathogenicity via the host immune evasion route. Experimentally validated immune epitopes were found for two out of three of these prognosis-associated SNVs. The epitopes containing the mutated forms exhibited a universally lower affinity to MHC-II than those with the reference forms (Figure 4A,B). Considering that various MHC-II genes have been implicated in the susceptibility of OPC across diverse global populations [42], the aggregation of these prognosis-associated SNVs likely enhances HPV16’s capacity to evade host immune surveillance.

## 4. Discussion

In this study, we conducted a comprehensive analysis of the therapeutic efficacy of TORS and the prognostic value of genomic variations in the HPV16 genome for patients with OPC.

With the development of robotic surgery, TORS has been widely used for patients with OPC. However, the indication of TORS for OPC remains unclear. The primary tumor (T) classification plays an important and decisive role in the choice of treatment for OPC patients. Our study demonstrates that OPC patients with a T1–T2 classification who received TORS had a better prognosis than those who did receive traditional resection. This might be because TORS can provide surgeons with a clear surgical field, especially in the deep and lower tumor margins. Thus, surgeons can accurately and completely resect the primary tumor with negative surgical margins. For patients with a T3–T4 classification, the tumor margins, and their visualization are limited due to the tumor’s bulky size within the anatomic subsite. These patients often have difficulty opening their mouths due to the muscle invasion of the lesion. Thus, it is difficult to completely remove the tumor via traditional surgery. With TORS, surgeons can observe the complete extent of the lesion with a small, flexible, and high-definition camera, which helps the surgeon to determine the lesion margin and improve the success rate of complete lesion resection. In addition, some scholars have proposed that tumor volume reduction via neoadjuvant chemotherapy or immunotherapy before surgery can also be applied to TORS treatment [43]. Nevertheless, TORS is still a new technology and needs to be carefully evaluated before being used for locally advanced OPC patients with a T3–T4 classification. Large-scale prospective studies are warranted to investigate the feasibility of TORS in patients with locally advanced OPC.

TORS’s effect in different stages of OPC patients was also analyzed. According to the 8th AJCC staging guidelines, the OPC patients with stages I–II were suggested to receive radical radiation, radical radiation plus chemotherapy, or surgery plus adjuvant therapy [44,45]. The main treatment principle for stage I OPC patients was to use a single treatment method to minimize the sequelae corresponding to treatment-related adverse reactions. Researchers are working on reducing radiation doses and omitting chemotherapy for stage II OPC patients [43,45]. The results of this study show a significant difference in 3-year OS between the TORS and non-TORS groups for OPC patients with stages I–II and stages III–IV indicating that TORS treatment could provide survival benefits. The survival benefits associated with TORS were consistently observed in both groups, reinforcing the potential of TORS as a superior therapeutic option in the management of OPC. These data advocate for a broader adoption of TORS in clinical practice, offering patients across various stages of OPC a better chance at not just survival but a higher quality of life post-treatment. Currently, the 8th AJCC staging guidelines recommend chemoradiotherapy or surgery plus adjuvant therapy for OPC patients with stages III–IV [44,45]. Therefore, as with OPC patients with a locally advanced classification (T3–T4), one must be cautious in assessing the tumor size, lymph node metastasis, and other aspects to determine whether OPC patients with stages III–IV are suitable for TORS treatment.

As for HPV-related OPC, previous studies have suggested that the overall prognosis of HPV^+^ OPC is better than that of HPV^−^ OPC [36,37], which was verified by this study. With the increased incidence of HPV^+^ OPC and different outcomes between HPV^+^ OPC and HPV^−^ OPC [46,47,48,49], the 8th edition of the AJCC staging guidelines differentiated OPC based on HPV status according to p16 overexpression. Due to the modification to the approach to N classification, many HPV^+^ OPC patients have been assigned lower stages relative to the previous criteria [44,50]. These changes have enabled improved OS discrimination, which is especially important in the research and exploration of treatment de-intensification [51,52]. OPC patients who received TORS alone (without adjuvant chemoradiotherapy) reported superior functional outcomes and quality of life (QOL), most probably reflecting the avoidance of the adverse effects of adjuvant therapy, including odynophagia, xerostomia, and oral thrush [53]. In addition, some HPV^−^ OPC patients were less sensitive to radiotherapy and chemotherapy [54], requiring higher-quality surgical treatment, while HPV^−^ OPC patients had better prognosis in the TORS group (Figure 1H), which not only emphasizes the advantages and importance of TORS in OPC treatment but highlights its critical role in treating HPV-negative patients who might not respond as well to other treatment modalities. These findings provide a strong impetus for the integration of TORS into treatment protocols for OPC, especially for HPV-negative cases, where conventional therapies may fall short.

Despite the better prognosis of HPV^+^ OPC, disease recurrence occurs in 10–25% of patients, the majority within 2 years after the initial diagnosis, but some within up to 5 years [50]; moreover, even with regular clinical examinations, the ability to detect disease recurrence and assess prognosis is limited. Thus, it is essential to explore clinically utilized prognosis-related markers for HPV^+^ OPC patients. In addition to the ability of sustained E6 and E7 expression to initiate tumorigenesis by involving the increased degradation of p53 and Rb [55,56,57,58,59], a high proportion of mutations in HPV^+^ OPC are thought to result from the off-target DNA-editing activity of apolipoprotein B mRNA-editing catalytic polypeptide-like enzymes (APOBEC3) [21]. A key consequence of APOBEC activity in HPV^+^ OPC appears to be the generation of oncogenic point mutations in PIK3CA [21,60], which has been associated with an increased risk of disease recurrence in HPV^+^ OPC patients receiving first-line chemoradiotherapy in trials exploring de-intensification approaches [61]. Furthermore, our study explored the relationship between genetic variations within the HPV genome and functional mutations in the corresponding proteins, heterogeneity in the clinical behavior and prognosis of HPV^+^ OPC patients. The three prognosis-associated SNVs were located within three regions of the HPV16 genome: the E1 (SNV1339A>G), E2 (SNV1950A>C), and L2 genes (SNV4298A>G). These mutations could induce structural and functional alterations in the corresponding proteins or even reduce their affinity for MHC-II binding, and thus affect the prognosis of HPV^+^ OPC patients. This highlights the potential value of these three SNVs as possible clinically utilizable prognosis-related markers. Moreover, licensed HPV vaccines are developed based on a virus-like particle (VLP) of the major papillomavirus capsid protein L1 and/or minor capsid protein L2 [62,63,64]. Considering the importance of the E1 and E2 genes in the carcinogenicity and prognosis of HPVs, the results of this study suggest that the E1 and E2 genes have great potential as targets for developing preventive and therapeutic HPV vaccines in the future.

While this study provides important insights, it is not without limitations. This study’s retrospective design failed to thoroughly investigate and explore the mortality cases, possibly introducing potential selection bias, given that the selection of OPC patients for TORS was based on comprehensive evaluations that included TNM classification, pathology, mouth-opening capacity, and general health status. Additionally, there was a lack of data completeness for the HPV infection statuses of OPC patients in the non-TORS group. Moreover, this study did not sufficiently address the impact of chemoradiotherapy and/or adjunct postoperative treatments on OPC patient outcomes. Furthermore, a comparative analysis regarding the efficacy of adjuvant chemoradiotherapy relative to TORS was absent. Finally, this study did not conduct experimental validation on the specific mechanism of HPV genome mutations affecting the prognosis of HPV^+^ OPC.

## 5. Conclusions

TORS showed good clinical application in OPC treatment, which can improve the prognosis of OPC patients, especially for those with a T1–T2 classification. The prognosis of HPV^+^ patients is better than that of HPV^-^ patients. The mutations in the HPV16 genome may affect the prognosis of OPC patients by changing the interactions and functions between proteins and reducing the MHC-II binding affinity to proteins, which can be used as clinical prognosis-related markers for HPV^+^ OPC.

## Figures and Tables

**Table 1:** Patient characteristics, overall and by study.

Characteristics	Overall (*n* = 265)	TORS Group (*n* = 130)	Non-TORS Group (*n* = 135)	*p*-Value
Age at diagnosis				0.357
≤45	35	14	21	
>45	230	116	114	
Gender				0.019
Male	222	108	114	
Female	43	22	21	
Smoking history				0.013
No	118	65	53	
Yes	147	65	82	
Drinking history				<0.001
No	77	46	31	
Yes	188	84	104	
HPV status				<0.001
Negative	86	62	24	
Positive	85	68	17	
Unknown	94	0	94	
Primary tumor (T) classification				0.009
1	65	31	34	
2	147	81	66	
3	21	6	15	
4	32	12	20	
Regional lymph node (N) classification				0.001
0	109	55	54	
1	43	23	20	
2	110	52	58	
3	3	0	3	
Distant metastasis (M) classification				0.234
0	262	130	132	
1	3	0	3	
Stage				0.001
I	57	36	21	
II	89	65	24	
III	26	3	23	
IV	93	26	67	
Postoperative adjuvant treatment				0.232
Yes	146	69	77	
No	119	61	58	

**Table 2:** The hazard ratios with 95% CIs and p-values of prognosis-associated SNVs.

Disease	SNV	MAF	Amino Acid Variant	Gene	Overall Survival			
					*p*-Value	Hazard Ratio	Lower 95% CI	Upper 95% CI
HPV^+^ OPC	SNV1339A>G	10.3%	Arg447Trp	E1	<1.0 × 10^−6^	6.08 × 10^3^	2.82 × 10^2^	1.31 × 10^5^
	SNV1950A>C	5.9%	Glu20Ala	E2	<1.0 × 10^−6^	3.10 × 10^4^	6.79 × 10^2^	1.41 × 10^6^
	SNV4298A>G	10.3%	Thr311Arg	L2	<1.0 × 10^−6^	5.23 × 10^2^	2.43 × 10^1^	1.13 × 10^4^
	≥1 high-risk SNVs	NA	NA	NA	<1.0 × 10^−6^	1.29 × 10^3^	7.67 × 10^1^	2.16 × 10^4^

## Data Availability

The data underlying this article are available herein and in the online Supplementary Material. Portions of the data from this article will be shared upon reasonable request from the corresponding author.

## Supplementary Materials

The following supporting information can be downloaded at https://www.mdpi.com/article/10.3390/jcm13154455/s1. Table S1: Clinical data; Figure S1: Flowchart illustrating the selection process of OPC patients for the non-TORS group; Figure S2: Flowchart illustrating the selection process of OPC patients for the TORS group; and Figure S3. Flowchart illustrating the selection process for the HPV16-WGS group.
