# Supplementary material for: Transoral Robotic Surgery and Human Papillomavirus Infection: Impact on Oropharyngeal Cancer Prognosis
Source: J Clin Med. 2024 Jul 30;13(15):4455. doi: 10.3390/jcm13154455 (PMC11313069; doi:10.3390/jcm13154455)
Supplement: Supplementary file 1 [file jcm-13-04455-s001.zip › jcm-3071977-supplementary.pdf]

Figure S1 non-TORS group

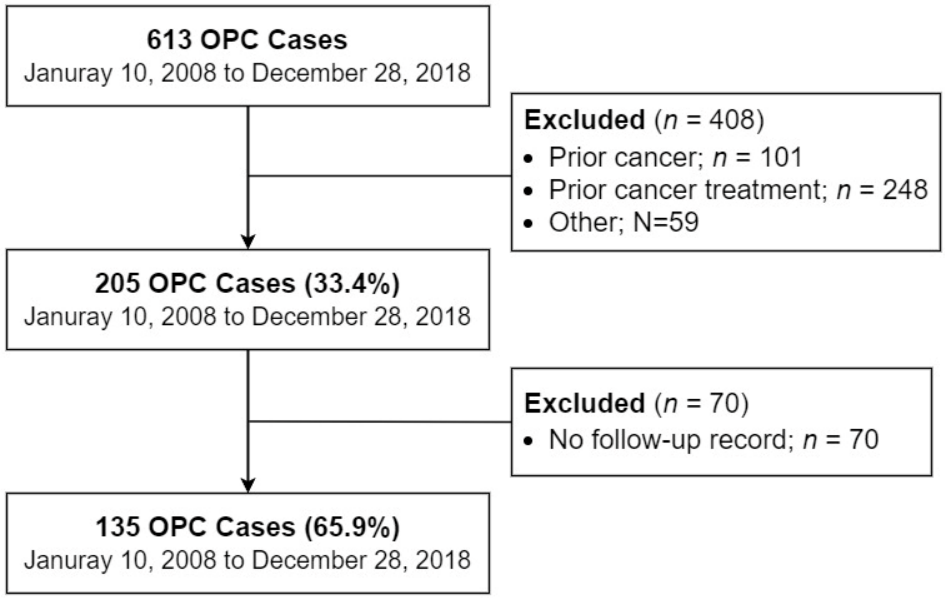

Figure S1. Flowchart illustrating the selection process of OPC patients for the non-TORS group.

Figure S2 TORS group

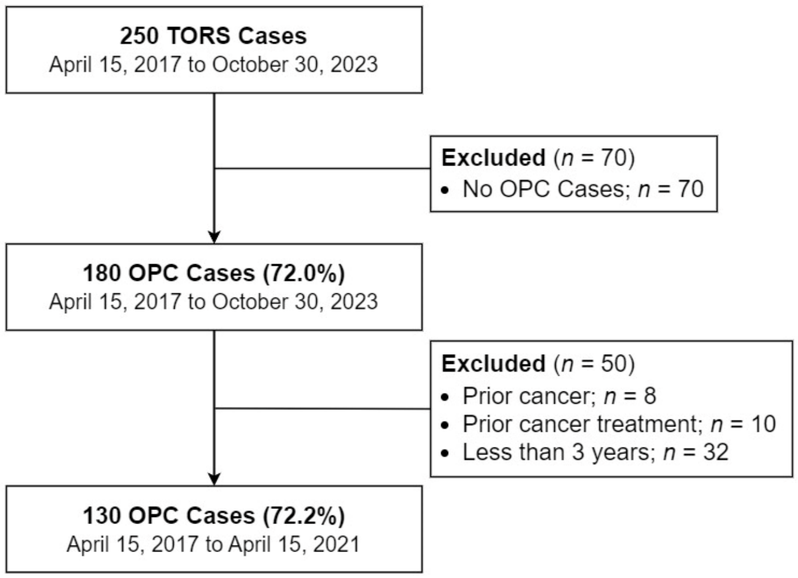

Figure S2. Flowchart illustrating the selection process of OPC patients for the TORS group.

Figure S3 HPV16-WGS group

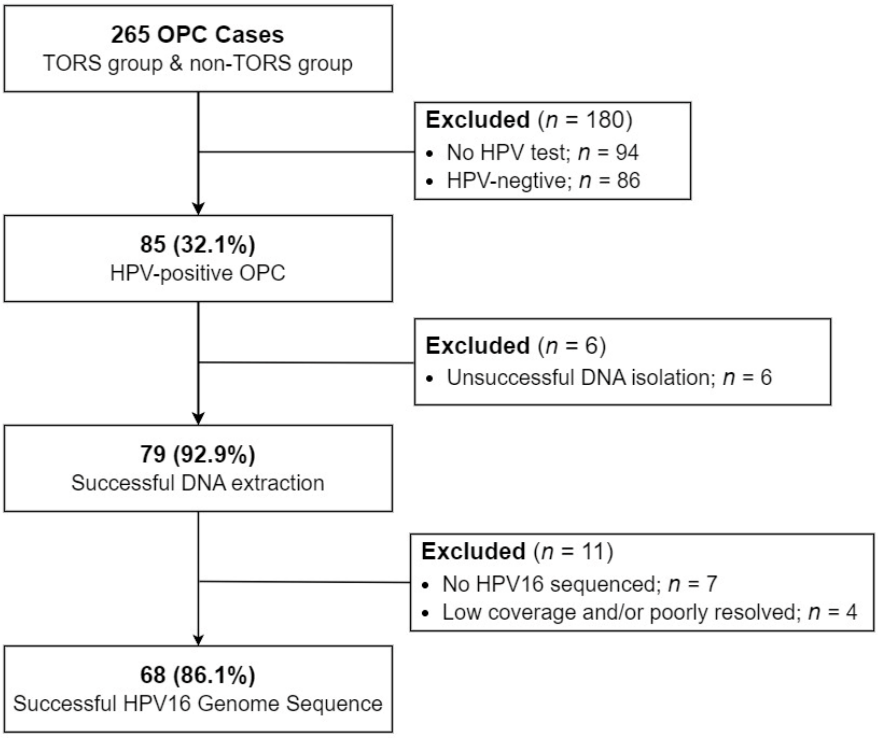

Figure S3. Flowchart illustrating the selection process for the HPV16-WGS group.
